# The effects of cognitive-behavioral group therapy for reducing symptoms of internet addiction disorder and promoting quality of life and mental health

**DOI:** 10.47626/2237-6089-2020-0010

**Published:** 2021-02-26

**Authors:** Seyyed Salman Alavi, Maryam Ghanizadeh, Mohammad Reza Mohammadi, Fereshteh Jannatifard, Sudeh Esmaili Alamuti, Malihe Farahani

**Affiliations:** 1 Psychiatry and Psychology Research Center Tehran University of Medical Sciences Tehran Iran Psychiatry and Psychology Research Center , Tehran University of Medical Sciences , Tehran , Iran .; 2 Kerman University of Medical Sciences Kerman Iran Kerman University of Medical Sciences , Kerman , Iran .; 3 Ministry of Education Iran Ministry of Education , Iran .; 4 Department of Psychology Allame Tabataba’i University Tehran Iran Department of Psychology , Allame Tabataba’i University , Tehran , Iran .

**Keywords:** Cognitive-behavioral therapy, internet addiction, quality of life, group therapy, psychopathology

## Abstract

**Introduction:**

Internet addiction disorder has reportedly become an important cause of health and social problems. The aim of this study was to investigate the effectiveness of cognitive-behavioral group therapy for internet addiction symptoms, quality of life, and mental health of students with internet addiction.

**Methods:**

This was a quasi-experimental study with pretest-posttest measures and a control group. The statistical population of the study consisted of all students at Tehran universities in the academic year of 2018-19. The target group was selected through an internet addiction test and a clinical interview using a targeted sampling method and was divided into experimental and control groups by randomization. The experimental group participated in fifteen 90-minute cognitive-behavioral group therapy sessions. Before, immediately after, and 3 months after the treatment, the internet addiction symptoms of both groups were evaluated to assess mental health with the IAT, quality of life (QOL), and SCL-90-R questionnaires. Data were analyzed with ANCOVA analysis using SPSS Statistics 20 software.

**Results:**

After treatment, cognitive-behavioral therapy groups showed reductions in internet addiction scores (p < 0.05). Results showed that the cognitive-behavioral group therapy was effective for improving quality of life (p < 0.05) and mental illnesses (p < 0.05) in students with internet addiction.

**Conclusions:**

Cognitive-behavioral group therapy can enhance awareness and mental health of students with internet addiction. Therefore, this intervention can be used as a beneficial treatment to reduce internet addiction symptoms and improve the condition of people with behavioral addictions such as internet dependency.

## Introduction

Internet addiction or internet dependency is a worldwide social issue and can exist at any age and in any social, economic, or educational strata. In some countries, such as Iran, behavioral addictions including internet addiction (IA) are considered a public health concern. ^1 , 2^

Internet addiction is considered a growing health concern in many countries of the world, with prevalence rates of 1-2% in Europe, ^3^ up to 24.5% in some Middle Eastern countries, ^4^ and 26.9% among Iranian students. ^5^ Internet addiction (IA) has been associated with psychiatric disorders such as anxiety, depression, personality disorders, and obsessive compulsive disorder (OCD), which cause a great deal of harm to quality of life. ^6^ Also, clinical research has demonstrated that internet addiction disorder is accompanied by loss of interests, decreased psychological functioning, social withdrawal, and heightened psychosocial distress. People with this disorder start to miss important deadlines at work, spend less time with their families, and slowly withdraw from their normal routines. They neglect social connections with their friends, coworkers, and communities; ultimately, their lives become unmanageable because of the internet. ^7^ They become consumed with their internet activities, preferring online games, chatting with online friends, or gambling over the internet, and ignoring family and friends in exchange for solitary time in front of the computer. While there are many studies assessing clinical features of persons with IA, ^6^ knowledge about the effectiveness of treatment sessions is limited.

Many treatment programs have been developed for patients with internet addition (IA), including pharmacotherapy ^8^ and psychological treatments such as cognitive-behavioral therapy. ^3 , 9 - 13^ Lee et al. reported that antidepressants are useful for treating internet addiction. ^14^ Han and Renshaw reported that bupropion may reduce depressive mood in patients with comorbid online addiction and depression. ^15^ However, the role of anti-craving properties of antidepressants should be further evaluated in the long-term and in controlled studies. ^16^

Cognitive-behavioral therapy (CBT) was performed once a week for 10 weeks and findings suggested that the sessions were effective to reduce internet addiction symptoms. ^17^

Young recommended a uniquely designed model to treat internet addiction, called cognitive-behavioral therapy for internet addiction (CBT-IA). ^9^ This study was the first to measure treatment outcomes using CBT-IA to treat cases of internet addiction. The results of several studies showed that psycho-behavioral therapy methods, such as group cognitive-behavioral therapy, are effective for treating internet addiction symptoms. ^18^

While many researchers have suggested treatment approaches to address internet addiction, little has been studied with relation to quality of life of internet addicts and therapy outcomes in terms of reduction of mental illnesses. The present study employed a research design to evaluate the utility and application of cognitive-behavioral group therapy (CBGT), consisting of 15 sessions to treat internet addiction symptoms. The outcome measure was appraised at the end of the 15 weekly therapy sessions and at 3 months after the treatment sessions.

Internet addiction disorder has been the subject of many studies since the last decade, but there is a need for an experimental or randomized controlled design for the topic, rather than cross-sectional studies. ^19^

However, studies that have been conducted were limited, their insufficient samples were not powerful enough to support the results of previous studies, and no standard clinical protocols have been developed to treat internet addiction. ^16^ Few studies focused on promotion of quality of life, treatment of IA symptoms, and reduction of mental illnesses such as anxiety, depression, somatization, and low self-esteem. ^12^ Moreover, developing preventive measures, especially for students, is of high importance. ^5^

### Research objectives

In this study, we aimed to collect the first data on the effectiveness of cognitive-behavioral group therapy for decreasing internet addiction symptoms such as craving for internet use. Furthermore, we also hypothesized that 15 sessions of therapy reduce some mental illnesses such as anxiety and depression. Lastly, we further expected that decreased craving for internet use and mental illnesses symptoms would be associated with promotion of quality of life, improvements in social relationships, and physical and psychological health in students.

## Material and methods

In this quasi-experimental study, data were collected from 50 internet addicts (25 in a control group and 25 in an experimental group) who consecutively presented at counseling centers for internet addiction at their universities (randomized clinical sample). These patients were selected from an initial clinical sample of about 60 people seeking treatment. Ten (16.3%) of these students were excluded because they did not meet the inclusion criteria: 5 students were normal internet users and 5 refused to participate. The entire sample (50 students) was asked to provide demographic data for scientific processing and provided written informed consent.

Inclusion criteria were as follows: 1) students aged 18-30 years (male or female); 2) diagnosed as an internet addict based on the standardized clinical interview and the internet addiction test (IAT) semi-structured interview for the assessment of internet addiction; and 3) willingness to participate in the study.

Exclusion criteria were as follows: 1) history of severe physical or psychological problems, including other addictive disorders, psychotic disorders, major depression, borderline personality disorder, or antisocial personality disorder based on the clinical psychologist’s view or observations and oral questioning); and 2) lack of participation in cognitive-group therapy sessions. Students who reported using medication for psychiatric disorders and those who were undergoing other psychotherapeutic treatments were also excluded from the study.

### Instruments

#### Data were collected using the instruments described below.

##### Internet Addiction Test (IAT)

The IAT is a self-report questionnaire based on the DSM diagnostic criteria for drug addiction and pathological gambling. It is a 20-item validated questionnaire with 5 components (Likert scale). After diagnosis of IA with the IAT, the severity of addiction was classified according to suggested scores of 20-49, 50-79, and 80-100 as normal user, moderate user, or severe internet user, respectively. Several studies have proved that Young’s internet addiction test has adequate psychometric properties (content and convergent validity, internal and external reliability). ^20 - 23^ The Persian version of IAT was used in this study. It has a Cronbach’s alpha of 0.89, and its reliability (test-retest) was 0.68 after 2 weeks. Therefore, it is a valid and reliable tool that can be used in psychological and psychiatric studies to screen normal internet users and internet addicts. ^24^

##### WHO-QOL-BREF

The WHO-QOL-BREF is a self-report questionnaire consisting of 26 items (with a 5-point Likert-type response scale), which appraises broad domains such as physical health, psychological health, social relationships, and the environment in adults over the age of 18. The WHO-QOL-BREF is a shorter version of the original tool that may be more convenient for use in large research studies or clinical trials. The WHO-QOL BREF has good internal consistency. Convergent validity between the WHO-QOL BREF and the Beck Depression Inventory (BDI) was statistically significant. Psychometrically, the WHO-QOL BREF is a valid and reliable instrument to measure quality of life in the general population. ^25^ Yousefy et al. reported that domain scores of the Persian version of the WHO-QOL-BREF demonstrate suitable internal reliability and criterion and discriminant validity. The physical health domain contributed most to overall quality of life, while the environment domain made the least contribution. Factor analysis yielded evidence of construct validity for the 4-factor model of the tool. The scores of all domains discriminated between patients and normal persons; therefore, it has adequate psychometric properties and is an adequate measure for evaluating quality of life at the domain level in an Iranian adult population. ^26^

##### Semi-structured interview

This semi-structured interview was performed by a clinical psychologist to diagnose behavioral addictions such as internet addiction. These interviews were performed by a psychologist trained in diagnosis of behavioral addiction in general and internet addiction disorder in particular. The criteria for diagnosing internet addiction were based on adapted DSM-5 criteria for gambling disorder and substance-related disorders ^27^ (e.g., preoccupation, loss of control, withdrawal, negative consequences, tolerance, and craving).

##### Symptom Checklist-90-Revised (SCL-90-R)

The SCL-90-R is a self-administered symptom questionnaire designed by Derogatis et al. A standardized Persian version exists and was used in this study. The Symptom Checklist consists of 90 items in total, divided into 9 symptom domains: somatization, obsessive-compulsive disorder (OCD), interpersonal sensitivity, anxiety, depression, phobic anxiety, hostility, paranoid ideation, and psychoticism. Each item covers one of the psychological symptoms with a Likert response scale with values ranging from ‘0 = no problem’ to ‘4 = very serious’ to describe the extent of symptoms that respondents had experienced during the last 2 weeks. In this study, the Persian version of the Symptom Checklist had a Cronbach’s alpha reliability of 0.95 and its split-half reliability was 0.88. ^28^

##### Cognitive-behavioral group therapy

Generally, cognitive-behavioral group therapy allows addicts to understand addictive feelings and actions while learning new coping skills and ways to prevent a relapse. Group therapy usually requires at least 3 months of treatment or approximately 15 weekly sessions. With internet addicts, the early stage of therapy should be behavioral, focusing on specific behaviors, cognitions, and situations in which impulse control disorder (ICD) causes the greatest difficulty. To determine the validity and reliability of the outcome checklist, mental health practitioners in the cognitive group therapy field evaluated the tool and conducted a pilot test. Ten cognitive-behavioral group therapists who were trained experts in CBT therapy were asked to evaluate the content of the CBT sessions and to comment on the clarity and suitability of the sessions. Before implementing the survey, a pilot test was also administered to 3 randomly selected students to assess the time required to finish the sessions, to clarify ambiguities and problems with the format if there were any, and to determine each session’s items. Thus, 15 sessions were held with the following titles ( Table 1 ).

**Table 1:** Summary of sessions for cognitive-behavioral group therapy

Sessions	Session titles	Goal(s)	Techniques	Practices
First session	Greeting and Introducing the Cognitive-behavioral Model	Description of the vicious cycle of thoughts, feelings and behaviors		
Second session	Cognitive model and its relation to “Internet Addiction”	Introducing stimulants or factors that lead a person to excessive use of Internet	Self-directed self-knowledge	The ways to promote self-awareness or self-knowledge The therapist will formulate a plan to achieve specific goals.
Craving and its coping methods				
Third session	Behavioral techniques to cope with craving	Changing patterns of behavior	Self-control	Doing practices such as Delayed Self-punishment
Fourth session	Cognitive techniques to cope with craving	Changing the way clients feel Changing patterns of thinking	Control of spontaneous thoughts Distinguishing healthy from unhealthy feelings	Distraction Identifying high-risk situations for excessive internet use Developing strategies for coping with and avoiding high-risk situations and the desire to use Conflict resolution skills Stopping thoughts Positive thought replacement
Fifth session	Treatment of depression and anxiety	Education about depression, anxiety and helping the client understand their symptoms as part of an illness	Identifying and engaging in enjoyable activities such as hobbies, social activities and exercise Exposure therapy	Helping clients to establish enjoyable activities in daily affairs Awareness of mood and challenging negative thoughts Doing pleasurable activities Learning to manage powerful emotions like anger, fear, or sadness
Sixth session	Relaxation training	Tension reduction	Techniques such as deep breathing, coping self-talk such as “I’ve done this before, just take deep breaths,” and distraction	Stress reduction techniques Deep breathing exercises Muscle relaxation Positive mental imagery
Seventh session	Training Activation and Planning	Cognitive restructuring or reframing	Activity scheduling and behavior activation	Replacement training Techniques for overcoming laziness
Eighth session	Lifestyle techniques	Improving lifestyle skills	Overcoming poor physical, psychological, and social skills	Attention to physical health Changing diet Doing exercise Controlling weight Avoiding drugs and cigarettes Expanding social relationships
Ninth & tenth sessions	Training Methods for Logical Analysis of Thoughts (two sessions)	Analysis of doubt and hesitation	Recording daily thoughts, emotions and behaviors	Understanding oneself, one’s world, and other people, Mindfulness
Eleventh session	Training problem-solving skills	Learning how to solve problems	Engaging in more social activities	Definition of the problem Generation of alternative solutions Evaluating and selecting an alternative solution Implementing and following-up on the solution
Twelfth session	Cognitive errors and their types	Description of cognitive errors	Methods of dealing with cognitive errors	Description of 12 cognitive errors
Thirteenth session	Methods to cope with cognitive errors	Description of distorted cognitions	Cognitive restructure	Identifying and modifying the thought process Focus on challenging unrealistic thinking.
Fourteenth session	Prevention of relapse	Managing symptoms or preventing internet addiction relapses	Helping the client to maintain and continue to quit or avoid excessive use of the internet	Training the necessary guides to deal with internet addiction
Fifteenth session	An overview of techniques presented at previous sessions, questions and answers		Review of techniques	

These cognitive-behavioral sessions were led by a clinical psychologist trained to diagnose and treat internet addiction. The content validity of sessions was checked and approved by 10 clinical and cognitive-behavioral psychologists.

## Procedures

In a randomized and quasi-experimental clinical trial with students from Tehran universities, 50 students with internet addiction disorder were selected based on IAT scores and a semi-structured interview. Pre-therapy and post-therapy assessments measured changes in students’ internet addiction symptoms, quality of life (QOL), and metal health (depression, anxiety, and somatization symptoms using the SCL-90-R). This design is well-documented in clinical and counseling literature on evaluating the effects of behavioral interventions and change over time. ^29^

All addicted internet users received standard behavioral treatment and were randomly divided into 2 groups. Written informed consent and assent were obtained from the students. Participants in the intervention group received cognitive-behavioral group therapy, while those in the control group only received standard individual psychotherapy. Both groups were evaluated before and after the intervention. All the sessions were completed by the students and the principle behavioral therapist. The sessions addressed symptoms related to problematic internet use, specifically, problematic online applications and strategies to control them. These sessions also focused on cognitive-behavioral issues and harm reduction for underlying factors contributing to pathological internet use, such as educational problems, problems with classmates or family members, or academic troubles, depending on each student’s unique situation. Seven participants in the experimental group and two in the control group dropped out of the study and the remaining participants all responded to questions promptly before and after the treatment.

Internet use was routinely assessed and treatment consequences were appraised after 15 sessions at a 3-month follow-up. Data were collected from both groups at the beginning of the study (T0) and after the final therapy session (15th session). The final stage was conducted approximately 3 months after the program was completed. Participants were asked to complete the questionnaires again in a self-report manner to follow the possible effects of the intervention.

## Data analysis

Data were analyzed for all participants who filled out both the baseline evaluation and at least one follow-up evaluation. Changes in primary and secondary outcomes after the intervention and after the 3-month follow-up were compared between groups using ANCOVA (with adjustment for baseline values).

## Ethics

This project was approved by Tehran University of Medical Sciences (grant number: (IR.TUMS.VCR.REC.1397.384), and the Iranian Registry of Clinical Trials (IRCT) approved the study procedures (IRCT20151128025270N2). All students were informed about the study’s goals and all of them provided informed consent.

## Results

**Figure 1:**
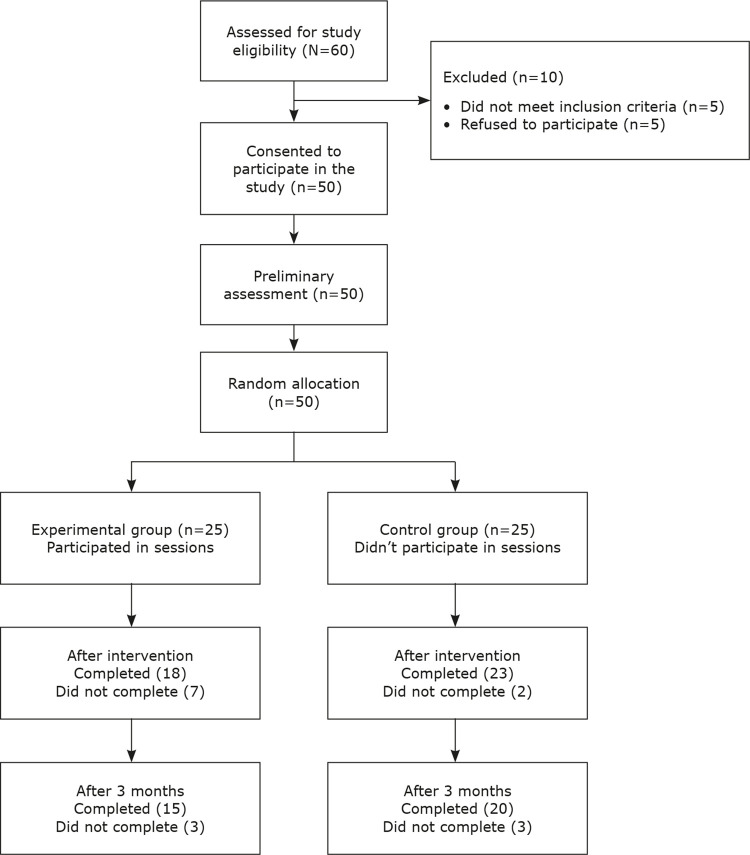
CONSORT (2010) flow diagram of participants.

Five of the 60 patients in the study did not meet the study’s inclusion criteria and 5 refused to participate. All of the 50 students who participated in the study completed the baseline assessment and were randomized into 2 groups ( Figure 1 ).

A total of 41 remaining students were evaluated. Demographically, 24.4% of the participants were female and 75.6% were male. The participants’ ages ranged from 18 to 28 years, 78% of them were single, 22% were married, 22% held a master’s degree or higher, 12.2% had a bachelor’s degree, and 65.8% were freshmen or sophomores.

The mean age of the participants was 21.7 years (SD = 4.2) for the experimental group and 21.04 years (SD = 3.7) for the control group. In terms of education, 66.7% of the participants in the experimental group were undergraduates and 33.3% were postgraduate students. Furthermore, 73% of the individuals in the control group were students studying towards a bachelor’s degree and 27% were students studying for a postgraduate degree. The demographic features of the participants are listed in Table 2 .

**Table 2:** Sociodemographic data on the participants

Sociodemographic variables	Experimental group	Control group
Gender		
Male	14 (77.8)	17 (73.9)
Female	4 (22)	6 (26.1)
Age (mean ± SD)	21.7±4.2	21.04±3.7
Age (range)	18-28 years	18-27 years
Marital status		
Single	15 (83.3)	17 (73.9)
Married	3 (16.7)	6 (26.1)
Housing situation		
With parents	4 (22.2)	8 (34.8)
Dormitory	14 (77.8)	15 (65.2)
Education		
Freshman or sophomore	13 (72.2)	14 (60.9)
Bachelor	2 (11.1)	3 (13)
MSc or higher	3 (16.4)	6 (26)

Data presented as n (%), unless otherwise specified.

SD = standard deviation.

The mean ± SD scores for internet addiction, quality of life, and SCL-90-R at the pretest, posttest, and follow-up stages are reported in Table 3 .

**Table 3:** Mean (SD) scores for internet addiction, quality of life and SCL-90-R at the pretest, posttest, and follow-up stages

	Groups	Experimental	Control
**Variables**			
Internet Addiction Symptoms			
b*		62.22±15,1	53.47±6.02
a**		40.1±10.9	49.3±8.6
f***		36.2±6.6	46.2±5.4
Total Score Quality of Life			
b		83.2±9.3	92.08±11.8
a		98.8±9.24	86.4±10.1
f		102.1±8.8	85.1±9.7
Total SCL-90-R score			
b		117.8±30.1	118.17±76.98
a		79.1±30.1	96.3±84.1
f		55.2±21.2	85.2±77.5

SD = standard deviation.

b* = before intervention;

a** = after intervention;

f** = follow-up stage.

Differences were observed between scores before and after the intervention and in the follow-up phase for quality of life, mental health, and internet addiction ( Table 3 ). The quality of life and mental health scores improved and the internet addiction scores decreased.

The results of ANCOVA showed significant differences in quality of life between the experimental and control groups at the posttest stage (p < 0.05) ( Table 4 ). Therefore, group cognitive-behavioral therapy could improve the health-related quality of life of students with IA.

**Table 4:** Results of analysis of covariance of mean scores for internet addiction, quality of life, and mental health at posttest

Variable/stage	Sum of squares	df	Mean squares	F	p value	Power
Internet addiction						
Pretest score	314.11	1	314.11	3.5	0.68	0.44
Grouping	1157.34	1	1157.34	13.01	0.001	0.94
Error	3380.01	38	88.94			
Total	88584	41				
Quality of life						
Pretest score	0.64	1	0.64	0.07	0.93	0.51
Grouping	1351.1	1	1351.1	13.88	0.001	0.95
Error	3698.86	38	97.33			
Total	351727	41				
Mental health						
Pretest score	69128.13	1	69128.13	3.4	0.71	0.44
Grouping	79473.5	1	79473.5	32.5	0001	1.00
Error	87973	38	2443.69			
Total	379596	41				

Most of the participants showed improvements after the 15 weekly sessions of cognitive-behavioral group therapy and the improvements remained at the end of the 3-month follow-up. An overwhelming majority of participants were able to manage internet addiction symptoms after the intervention program. Therefore, this program both addresses the internet use behavior and helps to reduce mental illnesses and improve quality of life.

## Discussion

This study was among the first to survey a therapeutic package (15 sessions) for students with internet addiction disorder.

The same measures were used to evaluate the outcomes at the end of the 15 weeks of cognitive-behavioral group therapy and 3 months later. The findings revealed that most of the participants were able to control symptoms of internet addiction as measured on the IAT and in the interview, which included the body of knowledge in IA (Internet addiction) and intervention plans. The findings of this study showed that symptoms of IA and mental disorders, such as depression, anxiety, and stress, decreased after attending the therapeutic sessions; hence, the study hypotheses are accepted.

A study has reported that scores for all the variables, including internet dependency, depression, anxiety and social anxiety, significantly decreased from pretest to posttest and had further decreased at follow-up. These results support the effectiveness of psychological programming to improve internet dependency symptoms. ^11^

In 2 other studies, reported in 2010 and 2011, the results showed that lifestyle patterns and dietary behaviors are also susceptible to changes caused by internet addiction, as are other physiological symptoms such as a lack of physical energy and weakened immunity. ^30 , 31^

In another two similar studies, in 2012 and 2013, the findings reported revealed that compulsivity, ruminative thoughts about internet, and cheating to access the internet were also reduced using CBT techniques. ^13 , 32^

Another study showed that CBT intervention programs for internet addiction were said to slowly rekindle offline relationships over time. ^10^ Also, internet use decreased in both groups while only school-based group CBT evinced improved time management skills and better cognitive, behavioral, and emotional symptoms. ^10^

Moreover, several studies revealed that cognitive distortions (errors) are most connected with internet addiction. ^9 , 33 , 34^ Therefore, cognitive-behavioral group therapy was developed to address these cognitive distortions.

Liu et al. argued that CBT psychotherapy made definitive and positive changes to depressive mood, somatization, anxiety, aggression, phobia and insecurity, paranoid ideation, and psychoticism. According to their research, each method of psychological psychotherapy, including CBT or sports intervention, had a significant effect on internet addiction and the symptoms of mental illnesses and sports intervention improved withdrawal symptoms. ^12^

Also, the review by Yeun et al. showed that psychological treatment could be used to treat internet addiction in children. ^19^ Park et al. evaluated the effects of group therapy in Korea ^35^ Oh et al. performed a meta-analysis on the effects of a program to prevent internet addiction and interventions in adolescents. ^36^

Therefore, since internet addiction is a compulsive behavior which causes serious impacts, with occupational and social problems, cognitive-behavioral group therapy has strong and positive effects on internet addiction (IA) and psychopathological symptoms.

Different paradigms of intervention can reduce the symptoms of internet addiction through promoting different aspects of interpersonal relationships and health issues, time management, and tolerance. Moreover, the different kinds of intervention may have different favorable effects on the psychological symptoms of somatization, social insecurity, OCD, depressive mood, anxiousness, aggression, paranoid ideation, and psychoticism, which means that cognitive group therapy may be administered to individuals with internet addiction who have different psychopathological symptoms.

Patients with internet addiction also had serious mental health problems, such as loneliness, low self-confidence or low self-esteem, anxiousness, and depression. ^37 - 40^ Adolescents spent more time browsing web sites or engaged in other addictive behaviors, which may lead to a lack of sleep at night and higher levels of fatigue. At the beginning of the 21st century, group therapy instructed group members how to recognize themselves and help themselves to improve social interaction and interpersonal relationships, and was capable of increasing adaptation and eliminating the symptoms of internet addiction. ^41^ A previous study identified useful effects of group therapy in adolescents with internet addiction, ^35^ which is in accordance with our results.

In this study, relaxation programming focused on the relationship with people with internet addiction who spent many hours on the internet. Relaxation programming can improve blood pressure and the oxygen supply to the brain, enhance the excitability of the cortex, and reinforce the flexibility of the nervous system, all of which promote functioning of the human body and psychological adaptability. Therefore, the relaxation intervention had several potential benefits over internet addiction treatment programs. In the present study, lifestyle changes improved anxiety, depression, aggression, social relationships, phobia ideation, paranoia, and psychotic symptoms. Apart from improving health issues, there were also improvements in time management skills, tolerance, compulsive internet use, and withdrawal symptoms after the therapy.

Another benefit of group therapy for treating internet addiction is the possibility of creating social connections among patients, as many of them are likely to be socially withdrawn. People who use the internet a lot, whether they like it or not, are socially isolated; therefore, they are unable to manage central dimensions of their lives due to their online preoccupation. Thus, they spend less time with their friends and family and have limited social activities. As the addiction continues, they prefer chats with online friends, or gambling on the internet. ^9^ This increases their isolation and so group therapy can provide an opportunity for them to free themselves of social isolation and improve the quality of their social relationships.

To sum up, most of the students in this study showed improvement after the 15 weekly sessions of cognitive-behavioral group therapy and improvements were sustained at the 3-month follow-up. During the treatment, participants improved their ability to communicate with peers in a social environment. Notably, the further improvements observed during the follow-up stage showed that the participants were able to distinguish between healthy and unhealthy or pathological internet use. These results suggest that the students were able to manage their internet use and expand social communications over time following the therapy.

Suggestions for further research include investigating the long-term effects of the model with a larger internet-addicted population using cognitive-behavioral therapy. As most of our adolescents and young people are experiencing psychological problems (e.g., anxiety, depression, ^42 - 44^ and communication and identity problems) ^45^ these cognitive-behavioral approaches will greatly improve their mental health. It should be noted that human behaviors are the result of many factors including social, cultural, and educational elements, ^46^ therefore all the results of this study have their own complexities that should be analyzed in more detail in future studies.

### Strengths

This study prepared a key experimental component of group CBT and helped in the expansion of empirically-based treatment plans that would suit the participants’ real needs. By doing so, it confirmed that successful therapy both explains internet addiction symptoms and helps students reduce anxiety, depression, or any other mental disorder, and increases social relationships to reduce pathological internet use.

### Limitations

Some limitations should be taken into account. This study was conducted on students who met the criteria for internet addiction, which means that the results should be generalized to those with a different level of education with caution. Moreover, to date, no studies have been conducted on the psychological effect of different types of psychotherapy or on comparing their quality and functions. Thus, use of other therapeutic options, such as spiritual therapy and drug therapy, along with CBT in future research is highly recommended. Furthermore, in this study, the sample size was small and the experimental group consisted of only 18 participants. Therefore, it would be useful to have more participants in future studies to collect more reliable data.

## Conclusions

The principal conclusion of this study was that cognitive-behavioral group therapy is an impressive treatment to improve lifestyle and reduce internet addiction. In this study, the behavioral package focused on modifying the individual’s thoughts, lifestyle, mental health, cognitive errors, emotional regulation, and problem-solving. Relaxation therapy and stress management may also be related to this study’s positive outcomes.

The nature of cognitive-behavioral group therapy makes it an effective instrument to treat those who cannot afford an expensive therapist. This therapy may be beneficial in treating other behavioral addictions, such as mobile dependency, and to decrease the symptoms of internet addiction. Further large studies with randomization or blinding techniques are also necessary to determine the effectiveness of different types of treatment, such as spiritual or life therapy, for internet addiction and psychopathological symptoms in Iran.
